# Changes of lip morphology following mandibular setback surgery using 3D cone-beam computed tomography images

**DOI:** 10.1186/s40902-016-0082-0

**Published:** 2016-10-05

**Authors:** Seung Jae Paek, Ji Yong Yoo, Jang Won Lee, Won-Jong Park, Young Deok Chee, Moon Gi Choi, Eun Joo Choi, Kyung-Hwan Kwon

**Affiliations:** 1 Department of Oral and Maxillofacial Surgery, College of Dentistry and Dental Hospital, Wonkwang University, Iksan, South Korea; 2 Department of Oral and Maxillofacial Surgery, Wonkwang Dental Research Institute, College of Dentistry, Wonkwang University, 460, Iksan-daero, Iksan, Jeollabuk-do 570-749 South Korea

**Keywords:** Three-dimensional evaluation, Class III malocclusion, Lip morphology, Mandibular setback surgery, Cheilion

## Abstract

**Background:**

The aims of this study are to evaluate the lip morphology and change of lip commissure after mandibular setback surgery (MSS) for class III patients and analyze association between the amount of mandibular setback and change of lip morphology.

**Methods:**

The samples consisted of 14 class III patients treated with MSS using bilateral sagittal split ramus osteotomy. Lateral cephalogram and cone-beam CT were taken before and about 6 months after MSS. Changes in landmarks and variables were measured with 3D software program Ondemand™. Paired and independent *t* tests were performed for statistical analysis.

**Results:**

Landmarks in the mouth corner (cheilion, Ch) moved backward and downward (*p *< .005, *p *< .01). However, cheilion width was not statistically significantly changed. Landmark in labrale superius (Ls) was not altered significantly. Upper lip prominence angle (ChRt-Ls-ChLt °) became acute. Landmarks in stomion (Stm), labrale inferius (Li) moved backward (*p* < .005, *p <* .001). Lower lip prominence angle (ChRt-Li-ChLt °) became obtuse (*p* < .001). Height of the upper and lower lips was not altered significantly. Length of the upper lip vermilion was increased (*p* =< 0.01), and length of the lower lip vermilion was decreased (*p* < .05). Lip area on frontal view was not statistically significantly changed, but the upper lip area on lateral view was increased and change of the lower lip area decreased (*p *> .05, *p *< .005). On lateral view, upper lip prominent point (UP) moved downward and stomion moved backward and upward and the angle of Ls-UP-Stm (°) was decreased. Lower lip prominent point (LP) moved backward and downward, and the angle of Stm-LP-Li (°) was increased. Li moved backward. Finally, landmarks in the lower incisor tip (L1) moved backward and upward, but stomion moved downward. After surgery, lower incisor tip (L1) was positioned more superiorly than stomion (*p* < .05). There were significant associations between horizontal soft tissue and corresponding hard tissue. The posterior movement of L1 was related to statistically significantly about backward and downward movement of cheilion.

**Conclusions:**

The lip morphology of patients with dento-skeletal class III malocclusion shows a significant improvement after orthognathic surgery. Three-dimensional lip morphology changes in class III patients after MSS exhibited that cheilion moved backward and downward, upper lip projection angle became acute, lower lip projection angle became obtuse, change of upper lip area on lateral view was increased, change of lower lip area decreased, and morphology of lower lip was protruding. L1 was concerned with the lip tissue change in statistically significant way.

## Background

Improvement in facial esthetics is one of the most important goals of orthodontic treatment and orthognathic surgery [1, 2]. To obtain the best results, treatment planning and the assessment of results should be performed on measurable three-dimensional reproductions of the face of the patients [3, 4]. However, the soft tissue response after mandibular setback osteotomies is subject to individual variation, and the predictability of soft tissue changes remains an important topic [5, 6].

Arnett and Bergman [7], Arnett et al. [2], and Proffit [8] emphasized the importance of esthetics in the frontal view, and orthodontists shifted the focus from the sagittal plane to the frontal plane when evaluating their patients and planning and assessing orthodontic treatment [9].

Researches have used lateral cephalogram to observe the aspect of transition regarding movement of soft tissue after surgery. However, it could result in inaccurate consequences in quantitative evaluation due to distortion of length, angle, and form by reflecting 3D structure in 2D plane [10]. Using laser scanner method can only measure change of soft tissue; therefore, in order to understand the change of skeletal structure, analysis of 2D radiograph should be enforced or 3D CT be re-filmed. Three-dimensional CT data, which can reduce the magnification and distortion errors of 2D radiographs [11, 12] and resolve the limitations of the 3D surface scanning system, have been used to analyze and measure the 3D structures [13–16].

Recent studies reported that in the smiles of the class III pretreatment group, both the upper and lower lips moved to an inferior position, and the upward movement of the upper lip and mouth corners was smaller, compared with those of the control group. And there were previous studies that lip morphology is different in patients with class III malocclusion when resting and smiling, and lip commissure is known to be inferiorly positioned than normal occlusion patient [17]. The morphology of lip and cheilion in class III patients may be shown in less esthetic.

In addition, there is no consensus about the changes of lip commissure related to the amount of mandibular setback. The position of lip commissure is known to be altered after orthognathic surgery. In recent study, upper lip projection angle (ChRt-Ls-ChLt °) became acute and lower lip projection angle (ChRt-Li-ChLt °) became obtuse [1, 18]. Change of labrale superius (Ls), cheilion, and labrale inferius (Li) had been debated. Jung et al. reported that the landmark in Ls was not altered significantly and cheilion, Li moved backward [19]. Lim et al. reported that the landmark in Ls, cheilion, and stomion moved backward and downward and Li moved backward [20]. Baik et al. reported that Ls, cheilion, and stomion and Li moved backward and downward [21]. In most papers, the width of the lip and the position of the upper lip were not altered significantly. And, the height of the lower lip was not altered significantly in some study. While, it was significantly decreased in other study [19–21].

Recent stuides reported and discussed about three-dimensional evaluation of facial soft tissue changes after mandibular setback surgery. They are consistent with the decrease of the upper lip prominence angle, increase of the lower lip prominence angle, and no change of the upper lip length. But, they are not consistent with the commissure position, the lower lip length, and so on. Also, there are few studies about the change of lip morphology according to mandibular setback surgery (MSS), according to the authors’ knowledge. We analyzed the lip morphology and change of lip commissure after mandibular setback surgery according to the amount of mandibular setback only (one jaw surgery) with comparison of preoperative and postoperative lip morphology.

## Methods

The samples consisted of 14 class III patients (seven males and seven females, mean age 20.3 ± 3.1 years, range 18 to 29 years) with Alphard-3030 cone-beam CT system (Asahi Roentgen Ind. Co., Ltd.) with the following parameters: 17-s scan time and a voxel size of 0.39 mm. And they had mandibular prognathism (ANB <0°, N-perpendicular Pog > 5 mm) and were conducted only MSS with bilateral sagittal split ramus osteotomy (BSSRO) by two surgeons (Table 1). Patients who had two jaw surgery or orthodontic treatment with premolar extraction or significant mandibular asymmetry (chin deviation >5 mm from facial midline) or lip incompetency or functional muscular problem were excluded. To evaluate the effect of the hard tissue changes on the soft tissue changes more accurately and to exclude the effect of the maxillary surgery on the soft tissue changes, the samples need to be limited to cases with MSS.

**Table 1:** Demographic data of the patients according to age, sex, genioplasty, time follow-up CT is taken, and mandible setback amount

Patient	Age	Sex	Genioplasty (mm)	Time follow-up CT is taken	Mandible setback (mm)
1	20	M	Reduction 3 mm	1 year	7
2	21	M	Advance 4 mm	2 years	4
3	18	F	Advance 4 mm	1 year	8
4	19	F	x	7 months	6
5	18	F	x	6 months	7
6	19	M	Advance 3 mm	6 months	6
7	18	M	x	10 months	11
8	19	M	Reduction 3 mm	1 year	7
9	19	F	x	6 months	11
10	20	F	Reduction 2 mm	6 months	4
11	24	F	x	1 year	7
12	29	M	Advance 3 mm	6 months	10
13	23	F	Advance 3 mm	1 year	7
14	18	M	Advance 5 mm	1 year	3

Cone-beam CT was taken immediately before (T1) and 6 months after MSS (T2) with centric occlusion, reposed lip, and natural head position and no face movement. We chose a period 6 months after surgery as the T2 stage because we reasoned that after this period, the facial soft tissue would be stable and any subsequent changes would be small enough to be ignored [22].

**Fig. 1:**
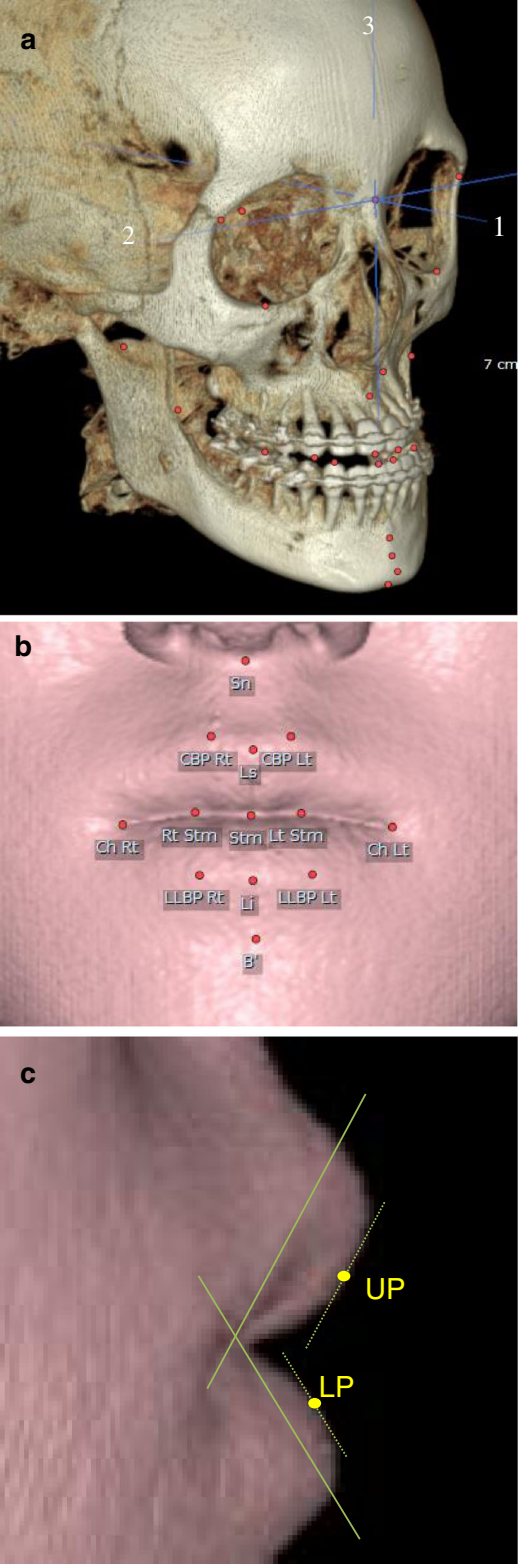
Landmarks and reference planes. **a** Reference plane: *1*, horizontal plane (Frankfort horizontal plane); *2*, coronal plane; *3*, sagittal plane. **b** Soft tissue landmark on frontal view. **c** Soft tissue landmark on lateral view

**Fig. 2:**
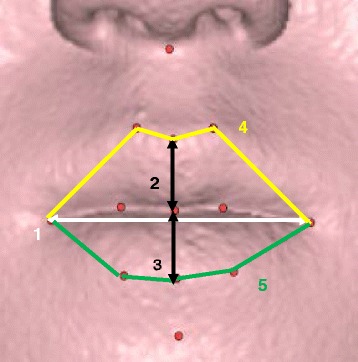
Linear variables. *1*, cheilion width; 2, upper lip height; 3, lower lip height; 4, upper lip vermilion length; and 5, lower lip vermilion length

**Fig. 3:**
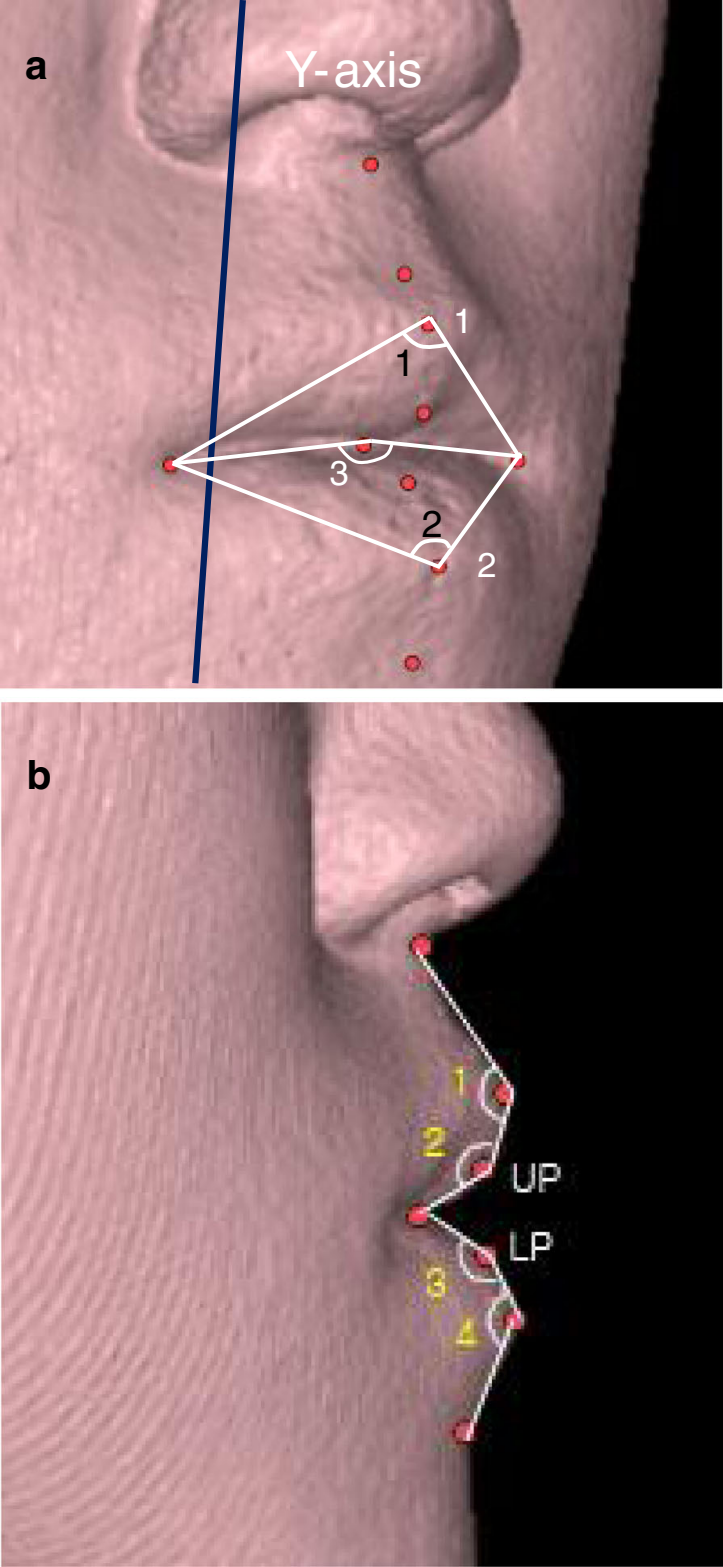
Angular variables. **a** *1*, UL prominence angle; *2*, LL prominence angle; 3, stomion prominence angle. **b** *1*, upper lip prominent angle (1) [Sn-Ls-UP °]; *2*, upper lip prominent angle (2) [Ls-UP-Stm °]; *3*, lower lip prominent angle (1) [Stm-LP-Li °]; *4*, lateral lower lip prominent angle (2) [LP-Li-Ln °]

The cone-beam CT raw data were reformatted into 3D views using 3D software program Ondemand™ (Cybermed Inc., Seoul, Korea). Reference plane was set by using landmark on orbitale, nasion, porion, and frontozygomatic suture that were not changed after operation. It was reoriented based on this landmark. Axes of *X*, *Y*, and *Z* was set in the horizontal, vertical, and anterior-posterior axis. Landmarks and reference planes are defined in Table 2 and Fig. 1. The 3D positions of the landmarks and five linear and seven angular variables at T1 and T2 stages were measured by a single operator using Ondemand 3D™ (Table 3 and Figs. 2 and 3). The extent of changes in the landmark and the linear and angular variables between the T1 and T2 stages were measured by a single operator using Ondemand program (Tables 3 and 4).

**Table 2:** Landmarks and reference planes used in this study

	Definition
Reference landmark	
Subnasale (Sn)	The midpoint of the angle at the columella base where the lower border of the nasal septum and the surface of the upper lip meet
Labrale superius (Ls)	The midpoint of the upper vermilion line
Cupid bow point (CBP)	The most elevated point of the philtrum on the upper vermilion border line
Labrale inferius (Li)	The midpoint of the lower vermilion line
Lower lip bow point (LLBP)	The breakpoint on the lower vermilion border line
Stomion (Stm)	The point at the midline of labial fissure between gently closed lips
Rt. Lt Stomion (Stm)	The breakpoint on the labial fissure line between gently closed lips
Cheilion (Ch)	The point located at each labial commissure
Soft tissue B point (B')	The deepest point on the facial midline, between the lower lip and chin
Lower incisor tip (L1)	The midpoint of the incisal edge of the right mandibular central incisor.
Upper lip prominent (UP)	A line parallel to the passing Ls to Stm touched the outer most point on upper lip from lateral view
Lower lip prominent (LP)	A line parallel to the passing Li to Stm touched the outer most point on upper lip from lateral view
Reference planes	
Horizontal plane	A plane constructed with the right orbitale, the right porion, and the left porion (Frankfort horizontal (FH) plane)
Coronal plane	A plane constructed with the right frontozygomatic suture (FSZ) and left FZS, perpendicular to the FH plane
Sagittal plane	A plane constructed with nasion, perpendicular to the FH and coronal planes

**Table 3:** Linear and angular variables of the hard and soft tissue

Reference mark	Definition
Linear variables (mm)	
Cheilion width	Distance between cheilion of the one side and the other side
Upper lip height	Distance between Ls and Stm
Lower lip height	Distance between Li and Stm
Upper lip vermilion length	Distance between Ch(Rt) and CBP Rt and Ls and CBP Lt and Ch(Lt)
Lower lip vermilion length	Distance between Ch(Rt) and LLBP Rt and Li and LLBP Lt and Ch(Lt)
Angular variables (°)	
UL prominence angle	Angle constructed among Ch, Ls, and Ch
LL prominence angle	Angle constructed among Ch, Li, and Ch
Stomion prominence angle	Angle constructed among Ch, Stm, and Ch
Lateral upper lip prominent angle 1	Angle constructed among Sn, Ls, and UP in lateral view
Lateral upper lip prominent angle 2	Angle constructed among Ls, UP, and Stm in lateral view
Lateral lower lip prominent angle 1	Angle constructed among Stm, LP, and Li in lateral view
Lateral lower lip prominent angle 2	Angle constructed among LP, Li, and Ln in lateral view

**Table 4:** Change of the hard tissue landmark (mm) after mandibular setback surgery

Landmarks	Δ(T2 − T1) (mm)		
	Mean	SD	*p* value
ΔB(z)	−5.05	2.84	0.000**
ΔL1(z)	−4.63	2.34	0.000**
Δpog(z)	−4.45	3.37	0.001**
ΔMe(y)	−1.20	1.34	0.014*

*Statistically significant difference between the groups (*P* <0.05)

**Statistically significant difference between the groups (*P* <0.01)

### Statistical analyses

We evaluated the clinical data of retrospective cohort study about 14 patients. The targets were patients who visited the Department of Oral and Maxillofacial Surgery at the Dental Hospital of Wonkwang University during January 2012~October 2015. An independent *t* test was used to compare the amounts of soft tissue changes with the amounts of hard tissue changes in T1 and T2. The correlations between the soft tissue and hard tissue movements in the horizontal and vertical planes were examined by Pearson correlation analysis. The horizontal ratios of the soft tissue to hard tissue changes in the groups were also compared. We conducted Shapiro-Wilks test to identify this normality test, and all variables had been normally distributed in significant probability over 0.05. A paired *t* test was performed to analyze the changes of 3D coordinate values and measurements between before and after the surgery. Statistical analysis was performed using Fisher’s exact test and Pearson correlation analysis and with SPSS ver. 12.0.

## Results

The samples consisted of 14 class III patients treated with MSS using bilateral sagittal split ramus osteotomy. The average periods when follow-up cone-beam CT was taken were about 10 months (range = 6 to 24 months) after operation. Five patients had not undergone genioplasty, three patients had undergone reduction genioplasty, and six patients had undergone advance genioplasty. The mean amount of setback at point B was 5.05 ± 2.84 mm (Table 1).

### Change of the hard tissue landmark (mm) between T2 and T1 stages

**Fig. 4:**
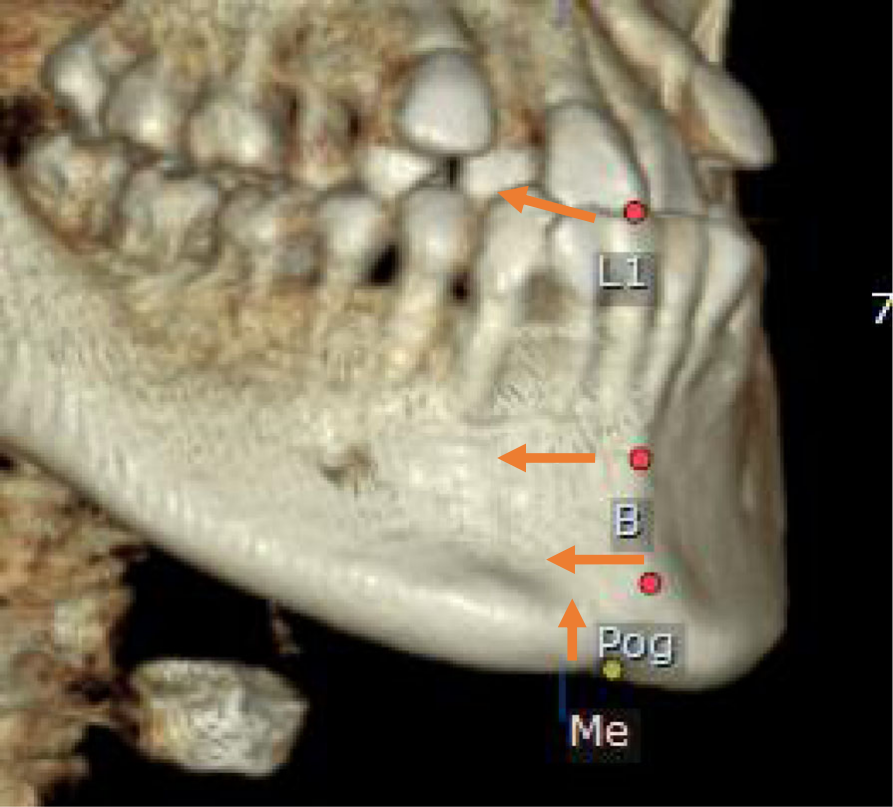
Change of the hard tissue landmark (mm) between T2 and T1 stages

No significant changes in the 3D position of maxillary hard tissue landmarks were found in either group. Anteroposterior (AP) changes in hard tissue landmarks on B point, pogonion, and L1 were significant in patients. Menton moved superior about 1.2 mm (Table 4 and Fig. 4).

### Change of cheilion, cheilion width, and lip prominence angle

Landmarks in the cheilion moved backward and downward (respectively 1.8 and 1.2 mm, *p* < .005 and *p* < .01). However, cheilion width was not statistically significantly changed. Landmark in labrale superius (Ls) were not altered significantly. Upper lip prominence angle became acute (ChRt-Ls-ChLt, 5° decreased, *p* < .005). Landmarks in stomion (Stm) and the lower lip (Li) moved backward (Stm, 1.8 mm; Li, 4.3 mm; *p* < .005, *p* < .001, respectively). Lower lip prominence angle became obtuse (ChRt-Li-ChLt, 9°, *p* < .001) (Tables 5 and 6 and Figs. 2 and 3).

**Table 5:** Change of cheilion position and cheilion width

Variables (mm)		Δ(T2 − T1)		
		Mean	SD	*p* value
Cheilion width	Ch Rt-Ch Lt	0.97	2.62	0.245
Cheilion AP position	ΔCh(z)	−1.85	1.6	0.003**
Cheilion vertical position	ΔCh(y)	−1.27	1.17	0.005**

**Statistically significant difference between the groups (*P* <0.01)

**Table 6:** Change of lip prominence angle

Lip area		Δ(T2 − T1)		
		Mean	SD	*p* value
Upper lip-related	ΔLs(z) (mm)	−0.18	2.00	0.765
	Ch(Rt)-Ls-Ch(Lt) (°)	−5.73	4.66	0.002**
Stomion-related	ΔStm(z)(mm)	−1.79	2.48	0.003**
	Ch(Rt)-Stm-Ch(Lt) (°)	−2.18	5.83	0.242
Lower lip-related	Δ(Li)(z)(mm)	−4.32	2.49	0.000**
	Ch(Rt)-Li-Ch(Lt) (°)	8.82	4.12	0.000**

**Statistically significant difference between the groups (*P* <0.01)

### Change in lip height, vermilion length, and the lip area

**Fig. 5:**
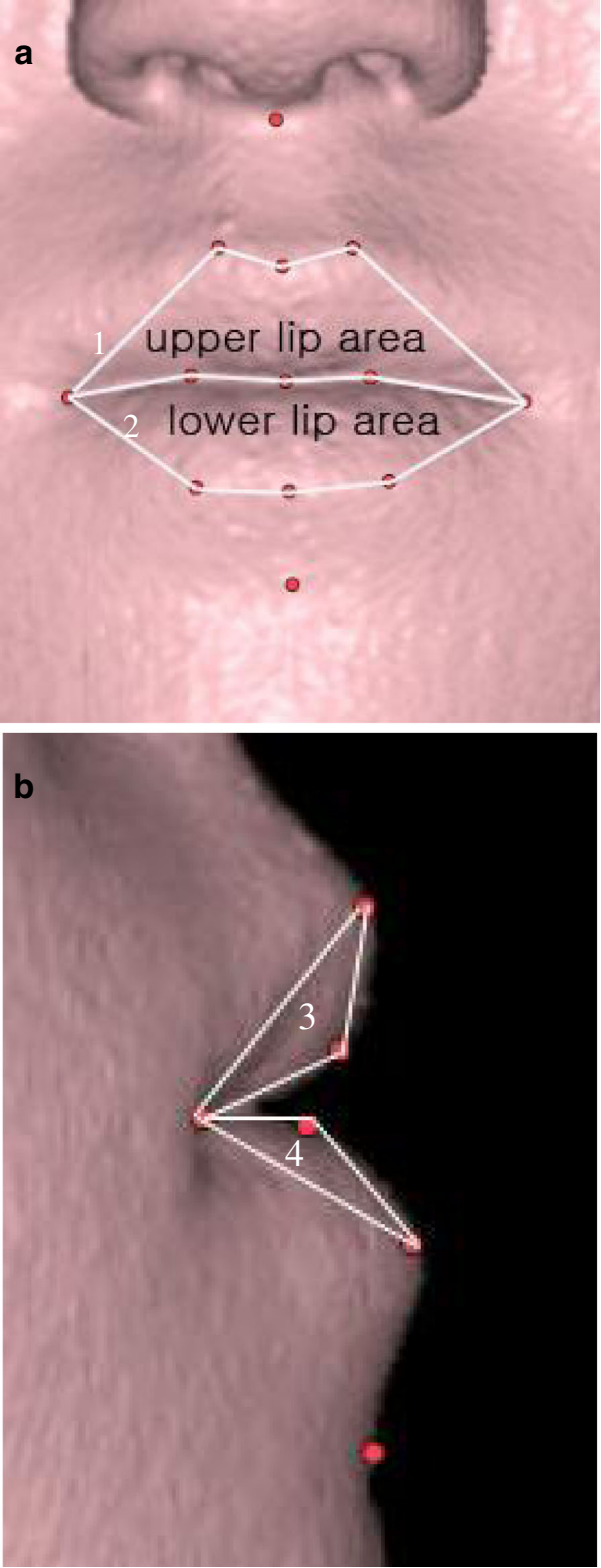
Lip areas of anterior and side views. **a** *1* upper lip area of frontal view; *2*, lower lip area of frontal view. **b** *3* upper lip area of lateral view; *4*, lower lip area of lateral view

The height of the upper and lower lips was not altered significantly. The length of the upper lip vermilion was increased (3.5 mm, *p* < .01) and the length of the lower lip vermilion was decreased (1.9 mm, *p* < .05) (Table 7 and Fig. 2). The lip area on frontal view was not statistically significantly changed, but the upper lip area on lateral view was increased and lower lip area decreased (upper lip area 6.3 cm^2^ increased, lower lip area 15.6 cm^2^ decreased, *p* > .05 and *p* < .005) (Table 9 and Fig. 5).

**Table 7:** Comparison of the change (from T1 to T2) in the upper lip height, lower lip height, upper lip vermilion length, and lower lip vermilion length

Variables (mm)		Δ(T2 − T1)		
		Mean	SD	*p* value
Upper lip height	Ls-Stm	0.76	1.42	0.105
Lower lip height	Li-Stm	−1.46	1.36	0.005**
Upper lip vermilion length	Ch(Rt)-CBP Rt-Ls-CBP Lt-Ch(Lt)	3.54	3.59	0.008**
Lower lip vermilion length	Ch(Rt)-LLBP Rt-Li-LLBP Lt-Ch(Lt)	−1.90	2.35	0.023*

*Statistically significant difference between the groups (*P* <0.05)

**Statistically significant difference between the groups (*P* <0.01)

### Change in the 3D linear angles of lip

**Fig. 6:**
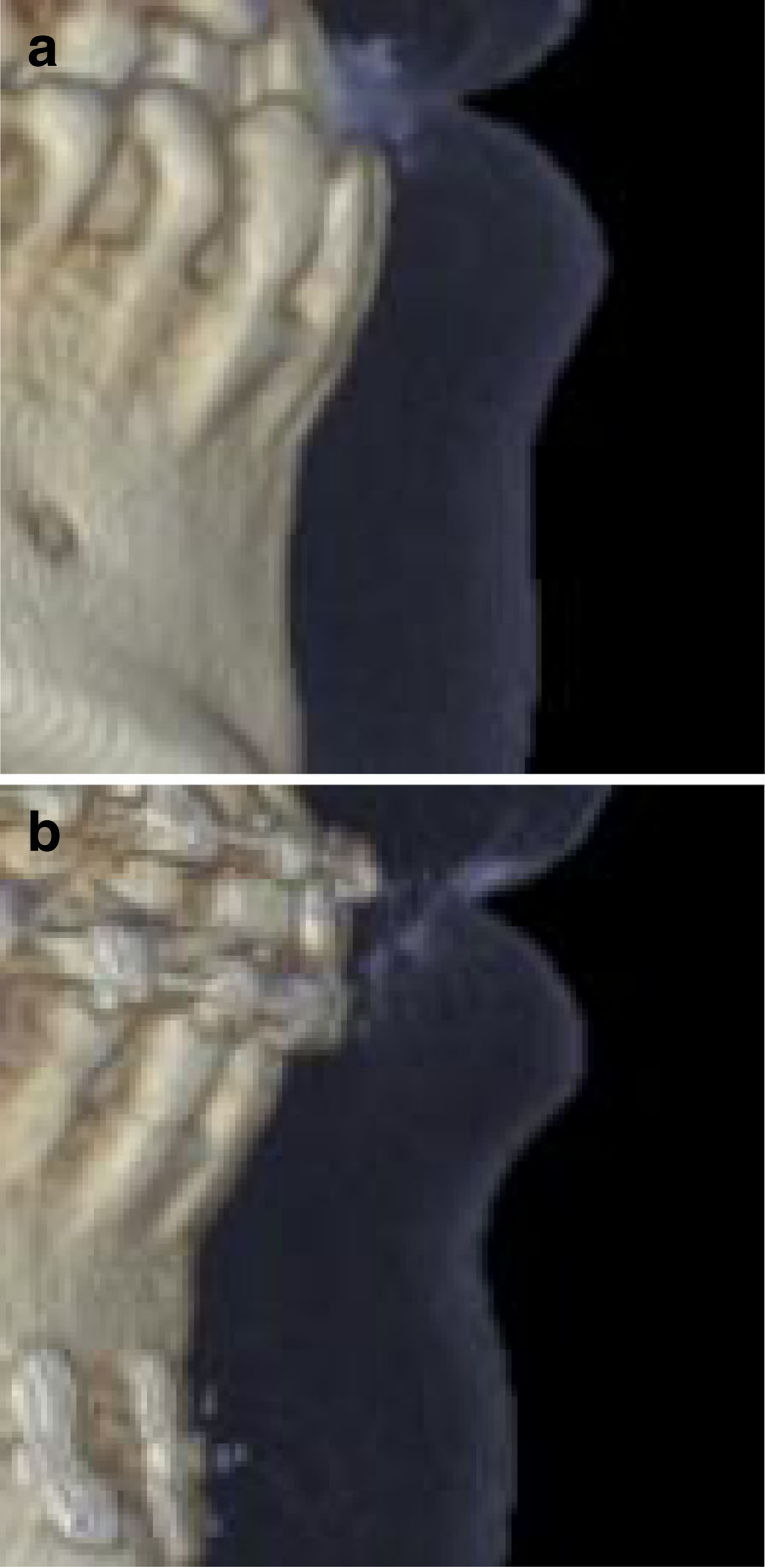
Change in the lower incisor tip and stomion. **a** Morphology in the lower incisor tip and stomion before MSS. **b** Morphology in the lower incisor tip and stomion after MSS

On lateral view, upper lip prominent point moved downward (1.24 mm, *p* < .05) and stomion moved backward and upward (1.79 and 0.63 mm, *p* < .005 and *p* = .103) and angle of Ls-UP-Stm (°) was decreased (−9.36°, *p* < .05). Lower lip prominent point moved backward and downward (−2.7 mm and −2.1 mm, *p* < .05 and *p* < .005) and the angle of Stm-LP-Li (°) was increased (6.43°, *p* =.065). Li moved only backward (−4.32, *p* < .001) and not downward. Finally, morphology of the lower lip was protruding (Table 8 and Fig. 6).

**Table 8:** Comparison of the change (from T1 to T2) in lip landmark and linear angles on lateral view

Variables (mm)		Δ(T2 − T1)		
		Mean	SD	*p* value
	UP(y) (mm)	−1.24	1.84	0.049*
	Stm(y) (mm)	0.63	1.17	0.103
	Stm(z) (mm)	−1.79	2.48	0.003**
	LP(y (mm))	−2.1	1.54	0.001**
	LP(z) (mm)	−2.7	3.44	0.025*
	Li(z) (mm)	−4.32	2.49	0.000**
1	Sn-Ls-UP (°)	−3.16	11.21	0.371
2	Ls-UP-Stm (°)	−9.36	10.75	0.016*
3	Stm-LP-Li (°)	6.43	10.29	0.065*
4	LP-Li-Ln (°)	5.19	12.59	0.201

*Statistically significant difference between the groups (*P* <0.05)

**Statistically significant difference between the groups (*P* <0.01)

### Change in the lower incisor tip and stomion

Landmark in the lower incisor tip (L1) moved backward and upward (−4.63 and 1.33 mm; *p* < .001 and *p* .084), but in stomion, the landmarks moved downward (0.63 mm; *p* = .103). Before the surgery, stomion was located higher than L1. After the surgery, L1 was positioned superiorly than stomion (−1.97 mm; *p* < .05) (Table 10 and Fig. 6).

### Correlation between the hard and soft tissue variables

The following is the result of Pearson coefficient analysis regarding the soft and hard tissue. The most influential landmark is L1, and it was related with cheilion’s backward and downward movement (*R* ^2^ = 0.67 and *R* ^2^ = 0.60; *p* < .05 and *p* = .05) and lower lip prominence angle (Stm-Y-Li °) (*p* =.057). The upper movement of menton is concerned with increase in stomion prominence angle (ChRt-Stm-ChLt °) (*R* ^2^ = 0.59; *p* < .05) (Table 11).

## Discussion

The lip is a structure positioned in the central part of the face, which has important influences on esthetic impression. After orthognathic surgery, the morphological change is inevitable so that accurate understanding about the alteration of soft tissue around lips is required.

Three-dimensional CT may be an effective tool for investigating the 3D changes in hard and soft tissues simultaneously in terms of direction and amount of movement information that 2D radiographs and 3D surface scanning systems cannot provide. If a better algorithm to combine the 3D laser or optical surface scanning and CT without distortional error is developed, it would be a great advance for the clinical research.

In our studies, these showed the same results as the previous studies. There was no change of lip width and decrease in upper prominence angle and lower lip vermilion, but increase of the lower prominence angle and the upper lip vermilion length showed the same results like the previous research [19–21].

### Change of cheilion, cheilion width and lip prominence angle

There have been controversies over mouth corner (cheilion position) in each study. In our study, landmarks in the mouth corner (cheilion, Ch) moved backward and downward after MSS (respectively 1.8 and 1.2 mm, *p* < .005 and *p* = .005). Lim and Baik et al. reported similar results, but Jung et al. reported different results that cheilion moved only backward [19–21]. The result of cheilion moving posteriorly is shown among the three studies. However, regarding the present study and the inferior movement of Lim and Baik’s one, the superior movement of Rafiqul after the surgery was the opposite result [17]. Further study is required for the inferior movement of cheilion.

Cheilion width was not statistically significantly changed. Landmark in labrale superius (Ls) were not altered significantly. It implies that muscle tonicity might not be changed within a short period of time [19]. Also, this means that an increase in the lip length between CBP and Ch was produced by stretching the soft tissues. Posterior and inferior movements of Ch were also observed in the 3D coordinates.

Upper lip prominence angle became acute (ChRt-Ls-ChLt, 5° decreased, *p* < .005). Landmarks in stomion (Stm) and the lower lip (Li) moved backward (Stm, 1.8 mm; Li, 4.3 mm; *p* < .005, *p* < .001, respectively). Lower lip prominence angle became obtuse (ChRt-Li-ChLt, 9°, *p* < .001) (Tables 5 and 6 and Figs. 2 and 3). These results were in accordance with the result of previous studies [19–21].

Before MSS, soft tissue stretching of the chin in the prognathic mandible can pull the upper and lower lips downward. When normal lip posture is achieved after MSS, normal convexity of the upper and lower lips can be restored. 3D soft tissue changes in class III patients after MSS occurred more in the Li than Ls with an increasing gradient. Because there was more backward movement of Li than Ls (4.3 vs. 0.2 mm, Table 6), the positions of the upper and lower lips were affected by the position of lower incisors in class III patients before MSS. However, after MSS, the upper and lower lips were under the influence of the upper incisors. The lower lip tension was reduced after MSS allowing for the formation of a better environment for lip sealing and eventually increase in lower lip prominence angle [20].

### Change in lip height, vermilion length and the lip area

The height of the upper and lower lips was not altered significantly. These results are in accordance with Jung et al. and Baik et al. [19, 21]. But the height of the lower lip is reported to change statistically significantly. They explain that the position of the lower lip would be influenced by the position of the upper incisors after MSS. In addition, lower lip tension could be reduced after MSS and eventually resulted in decrease of the lower lip height and lower vermilion height, which is consistent with Gjørup and Athanasiou [20, 23].

However, the vertical change of the soft tissue after surgery is still difficult to predict. Robinson et al. and Jung et al. reported that changes in the soft tissue did not closely follow those in the hard tissue in the vertical plane compared with the anteroposterior and transverse planes [19, 20, 24].

The length of the upper lip vermilion was increased (3.5 mm, *p* < .01) and the length of the lower lip vermilion was decreased (1.9 mm, *p* < .05). After MSS, there is a possibility of decrease in lower lip tension, which would allow for replacement of the upper lip over the lower lip and eventual opposing change in the vermilion border length [20].

On frontal view, the lip area was not statistically significant changed, but the upper lip area on lateral view was increased and the lower lip area decreased after MSS (6.3 cm^2^, *p* = .078, –15.6 cm^2^, *p* < .005). This is due to the cheilion which moved backward and downward and labrale inferius which moved backward. These changes make the facial profile more esthetic in class III patients (Table 5, 9 and Fig. 5).

**Table 9:** Comparison of the change (from T1 to T2) in the lip area

Lip area (cm^2^)		Δ(T2 − T1)		
		Mean	SD	*p* value
Upper lip	Lateral view	6.28	10.59	0.078*
	Frontal view	8.21	45.18	0.560
Lower lip	Lateral view	−15.57	10.33	0.001**
	Frontal view	−12.69	41.46	0.334

*Statistically significant difference between the groups (*P* <0.05)

**Statistically significant difference between the groups (*P* <0.01)

### Change in the 3D linear angles of lip

On lateral view, the upper lip prominent point moved downward (1.24 mm, *p* < .05) and stomion moved backward and upward (1.79 and 0.63 mm, *p* < .005 and *p* = .103) and the angle of Ls-UP-Stm (°) was decreased (−9.36°, *p* < .05) (Table 8). The lower lip prominent point moved backward and downward (−2.7 and −2.1 mm, *p* < .05 and *p* < .005) and the angle of Stm-LP-Li (°) was increased (6.43°, *p* .065) (Table 8). Li moved only backward (−4.32, *p* < .001) and not downward. Finally, morphology of the lower lip was protruding (Table 8 and Fig. 6).

The eversion of the lower lip became distinct, and a more natural lip line was formed. After the surgery, the outcome of the lower lip eversion is shown in other studies as well [17, 19–21]. Three-dimensional optical surface scans could measure the magnitude of change and show a significant eversion of the lower lip, which was reported by Hershey and Smith [23].

In that study, using the thin-plate spline analysis, the forward movement of stomion inferius was shown, while labrale inferius was moving backward, displaying the eversion of the lower lip and increasing the lower vermilion border in most class III cases. This was because the relaxation of the lower lip after the tension caused by the lower teeth and the alveolar bone was eliminated with a mandibular setback, eventually eliminating lip incompetence and forming a better lip seal.

### Change in the lower incision tip and stomion

Landmarks in the lower incisor tip (L1) moved backward and upward but, in stomion moved downward. After surgery, L1 was positioned superiorly than stomion (−1.97 mm, *p* < .05) (Table 10 and Fig. 6). According to Robinson, Ls and Li moved inferiorly, even though almost all hard tissue landmarks had moved superiorly. This was because the free ends of the upper and lower lips interfered with the contacting incisor surface and the opposite lips recovered by moving downward [24].

**Table 10:** Comparison of the change (from T1 to T2) in lower incisor lip and stomion

	ΔT1			ΔT2			Δ(T2 − T1)		
	Mean	SD		Mean	SD		Mean	SD	*p* value
ΔL1(y) (mm)	82.35	6.48	0.000	82.84	5.62	0.000	−1.33	2.30	0.084
ΔStm(z) (mm)							−1.79	2.48	0.003**
ΔStm(y) (mm)	84.17	7.40	0.000	82.99	6.24	0.000	0.63	1.17	0.103
ΔStm(y) − ΔL1(y) (mm)	1.81	2.84	0.060	−0.15	2.4	0.834	−1.97	2.17	0.013*

*Statistically significant difference between the groups (*P* <0.05)

**Statistically significant difference between the groups (*P* <0.01)

### Correlation between the hard and soft tissue variables

The following is the result of Pearson coefficient analysis regarding the soft and hard tissue. The most influential landmark is L1, and it was related with cheilion posterior movement (*p* < .05 and *p* = .05) and lower lip prominence angle (Stm-Y-Li °) (*p* = 0.057). The upper movement of menton is concerned with increase in stomion prominence angle (ChRt-Stm-ChLt °) (*p* < .05) (Table 11). These changes may be a contributing factor that the lower lip makes eversion and is more esthetic. Perhaps, decrease of muscle tension might change the prominent angle of the upper lip and lower lip at lateral view. It might make the form of the lip esthetically and eversion.

**Table 11:** Correlation between the anteroposterior (AP) and vertical (V) changes in the hard and soft tissue variables

Correlation variables		*R* ^2^	*p* value
ΔB(z) vs.	ΔLi(z) (mm)	0.737	0.010**
ΔL1(z) vs.	ΔCh(y) (mm)	0.602	0.050*
	ΔCh(z) (mm)	0.671	0.024*
	ΔLi(z) (mm)	0.848	0.001**
	ΔStm(z) (mm)	0.745	0.008**
	ΔStm-Y-Li (°)	0.588	0.057*
ΔPog(z) vs.	ΔLi(z) (mm)	0.629	0.038*
ΔMe(y) vs.	ΔCh Rt-Stm-Ch Lt (°)	0.646	0.032*

*Statistically significant difference between the groups (*P* <0.05)

**Statistically significant difference between the groups (*P* <0.01)

Correlations and ratios of the anteroposterior and vertical changes between the hard and soft were examined. Pearson correlation analysis was done. Ratio (*R* ^2^) means amount of change in the soft tissue/amount of change in the hard tissue. Positive (+) value, the change with the same direction; negative (−) value, the opposite direction

Lip commissure was seemed to be positioned higher in postoperative phase than in preoperative phase. It may be due to increase of lip thickness. Finally, lip morphology seems to be improved in the view of esthetics after orthognathic surgery (one jaw) for mandibular prognathism.

After mandibular setback, the position of the lip commissure tended to be higher, and the amount of 1.27 mm was not a few changes. This amount of changes was statistically significant.

Because of the limited number of the included patients in this study, authors could not analyze how much genioplasty affects the changes of lip morphology. Afterwards, there would be a research more focus on the change in lip morphology according to genioplasty or non-genioplasty group based on significant number of patients. Two-jaw surgery is more influencing on the muscle and soft tissue than one-jaw surgery, and further research on the lip change is needed. Further researches are needed to be followed on this topic, and it would help us to predict the changes of lip morphology according to the orthognathic surgery.

## Conclusions

Three-dimensional evaluation using 3D cone-beam CT is a valuable tool for assessing the postsurgical changes in movements and allows assessment of changes that 2D imaging modalities do not. The 3D soft tissue changes in class III patients after MSS did exhibit increased gradients from the upper lip and the lower lip to the chin and from the midline (Stm) to the lateral area (Ch).
